# Identification of somatic and germ-line DICER1 mutations in pleuropulmonary blastoma, cystic nephroma and rhabdomyosarcoma tumors within a DICER1 syndrome pedigree

**DOI:** 10.1186/s12885-017-3136-5

**Published:** 2017-02-21

**Authors:** Lorena Fernández-Martínez, José Antonio Villegas, Íñigo Santamaría, Ana S. Pitiot, Marta G. Alvarado, Soledad Fernández, Héctor Torres, Ángeles Paredes, Pilar Blay, Milagros Balbín

**Affiliations:** 1 Laboratorio de Oncología Molecular, Instituto Universitario de Oncología del Principado de Asturias (IUOPA), AGC Laboratorio de Medicina, Hospital Universitario Central de Asturias (HUCA), Oviedo, 33011 Spain; 2 Unidad de Oncología Pediátrica, AGC Pediatría, Hospital Universitario Central de Asturias (HUCA), Oviedo, Spain; 3 Servicio de Anatomía Patológica, Hospital Universitario Central de Asturias (HUCA), Oviedo, Spain; 4 Unidad de Cáncer Familiar, Servicio de Oncología Médica, Hospital Universitario Central de Asturias (HUCA), Oviedo, Spain

**Keywords:** DICER1 mutations, DICER1 syndrome

## Abstract

**Background:**

DICER1 syndrome is a pediatric cancer predisposition condition causing a variety of tumor types in children and young adults. In this report we studied a family with two relatives presenting a variety of neoplastic conditions at childhood.

**Methods:**

Germ-line mutation screening of the complete coding region of the *DICER1* gene in genomic DNA from the proband was performed. The presence of somatic *DICER1* mutation and further alterations in driver genes was investigated in genomic DNA obtained from available tumor samples.

**Results:**

A nonsense germ-line mutation in *DICER1* causing a truncated protein at the IIIb domain level was identified segregating within a family including two affected relatives who developed in one case cystic nephroma and pleuropulmonary blastoma, and rhabdomyosarcoma and multinodular goiter in the other. Additional *in trans DICER1* missense somatic mutations in the IIIb DICER1 domain were found both in the cystic nephroma and in the rhabdomyosarcoma, suggesting that neoplasms in this family might arise from the unusual two-hit mechanism for DICER-derived tumorigenesis in which after the presence of a truncated constitutive protein, a neomorphic DICER1 activity is somatically adquired. Additional genetic alterations, such as TP53 mutations, were identified in the rhabdomyosarcoma.

**Conclusions:**

Besides *DICER1* loss of standard activity, oncogenic cooperation of other genes, as mutated *TP53*, may involve developing higher grade tumors within this syndrome. Given the broad clinical spectrum that may arise, genetic counseling and close surveillance must be offered to all family members at risk of DICER1 syndrome.

## Background

Germ-line mutations in *DICER1* have been described in the so-called DICER1 syndrome, a pleiotropic pediatric cancer predisposition condition causing a variety of tumor types in children and young adults, including pleuropulmonary blastoma (PPB), cystic nephroma (CN), rhabdomyosarcoma (RMS), multinodular goiter, ovarian Sertoli-Leydig cell tumor and other neoplastic conditions. DICER1 is a multidomain protein, containing two endoribonuclease III domains. In the majority of cases, germ-line mutations are nonsense, frameshift or splice-site mutations leading to premature truncation of the protein, resulting in loss of RNAseIII function [1–3]. RNA processing endoribonucleases are required for the biogenesis of microRNAs (miRNAs), cleaving precursor miRNAs into mature miRNAs which, in turn, post-transcriptionally regulate messenger RNA expression [2]. Disregulation of miRNAs is implicated in several human diseases, as they participate in many different biological processes. Thus, mutations in *DICER1* have the potential to affect many biological functions and originate different phenotypes*.* In this communication, germ-line and somatic mutations in *DICER1* are reported within a family with two relatives presenting a variety of neoplastic conditions at childhood.

## Methods

### Subjects

Our studied pedigree comprised 8 individuals. All subjects or their parents/legal guardians gave written informed consent for genetic research studies and peripheral blood samples were taken. Available frozen tumor tissue samples were obtained from HUCA Tumor Bank. Written informed consent for sample banking and research use was obtained at the time of the surgery.

### DNA, RNA and cDNA samples

DNA from peripheral blood and tumor tissues was extracted using DNAzol (Molecular Research Center, USA), following manufacturer’s instructions. RNA was obtained from frozen CN and ERMS frozen tissue samples with Tri-reagent® (Ambion). cDNA was synthesized with RNA ImProm-II Reverse Transcriptase (Promega Corporation, Madison, WI, USA), following manufacturer’s instructions.

### Mutational analyses

Individual coding exons of the *DICER1* and *TP53* gene including flanking intronic regions were amplified by PCR. Primer sequences are available on request. Purified sequence reactions were resolved on a capillary automated Sanger sequencing (ABIPrism310, Applied Biosystems, Thermo Fisher Scientific). GenBank reference sequence accession numbers were NM_177438, NG_016311, and NP_803187 for *DICER1* and NG_017013.2, NM_000546.4 for *TP53* gene.

Hot spot mutations in *KRAS, NRAS, EGFR, PIK3CA* and *BRAF* were sought by real-time quantitative allele-specific PCR amplification using commercial kits (RAS Mutation Screening Panel, Entrogen, USA; *therascreen* EGFR RGQ PCR Kit V2, Qiagen, UK; *cobas* PIK3CA Mutation Test, Roche, USA; *cobas* 4800 BRAF V600 Mutation Test, Roche, USA), following manufacturer’s instructions. Deletions or amplifications in *PDGRFA, TP53, CDKN2A, CDK4, RB1, EGFR, PTEN,* and *MMDM2* genes and in the chromosomal regions 1p and 19q were studied by MLPA (Multiplex Ligation-dependent Probe Amplification) using commercial kits (P0471, P088, and P105 probemixes, MRC-Holland, The Netherlands), following manufacturer’s instructions.

## Results

### Case report

**Fig. 1:**
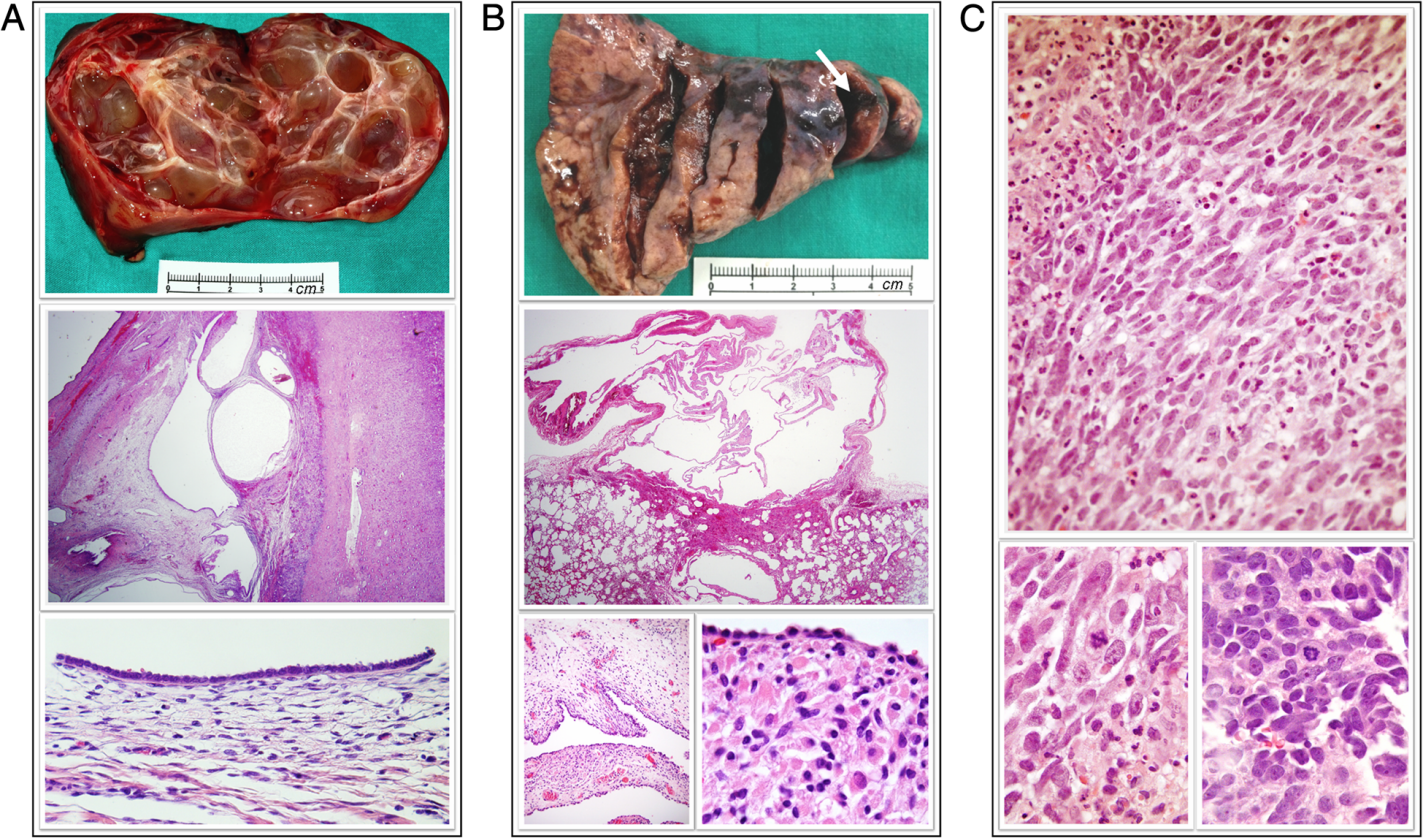
Morphologic study of the reported tumors. **a**. Left nephrectomy from the proband. Top: Gross morphology image of the resected kidney presenting a thin-wall multicystic parenchyma. Middle: Medium power view of a haematoxylin and eosin staining (HE) showing multiple cysts of different size. Scarce renal tissue is preserved. Bottom: Higher power view of the cysts surface showing a single layer of flattened/cuboidal cells with well oriented nuclei, acidophilus cytoplasm, and without atypia. **b**. Left lower lobectomy from the proband. Top: Gross morphology image of the resected lobe. An arrow is pointing to the cystic lesion of 12 mm largest size. Middle: At histological level, a unique benign cystic lesion was identified (HE). Bottom left: A single cubic-cell layer lining the epithelium was observed in most of the cystic lesion. Bottom right: Only very few and isolated clumps of primitive mesenchymal cells and small clusters of cells with rhabdomyomatous differentiation were found along the whole tissue sample. **c**. Histological images of the rhabdomyosarcoma from the proband’s cousin. Top: HE staining showing dense neoplastic cell proliferation in a solid-storiform pattern. Bottom: Intense pleomorphism is observed, with fusiform-oval cells and common mitotic figures, with focal anaplastic cells

Index case was a 2-year-old girl presenting a cystic nephroma at 11 months of age that required surgical resection of her left kidney. A computed tomography of the chest revealed a cystic mass in the left lower lobe adjacent to the diaphragm. At 14 months of age, a left lower lobectomy was performed and a pathological diagnosis of type I PPB was established (Fig. 1a and b).

### Germ-line mutational analysis

**Fig. 2:**
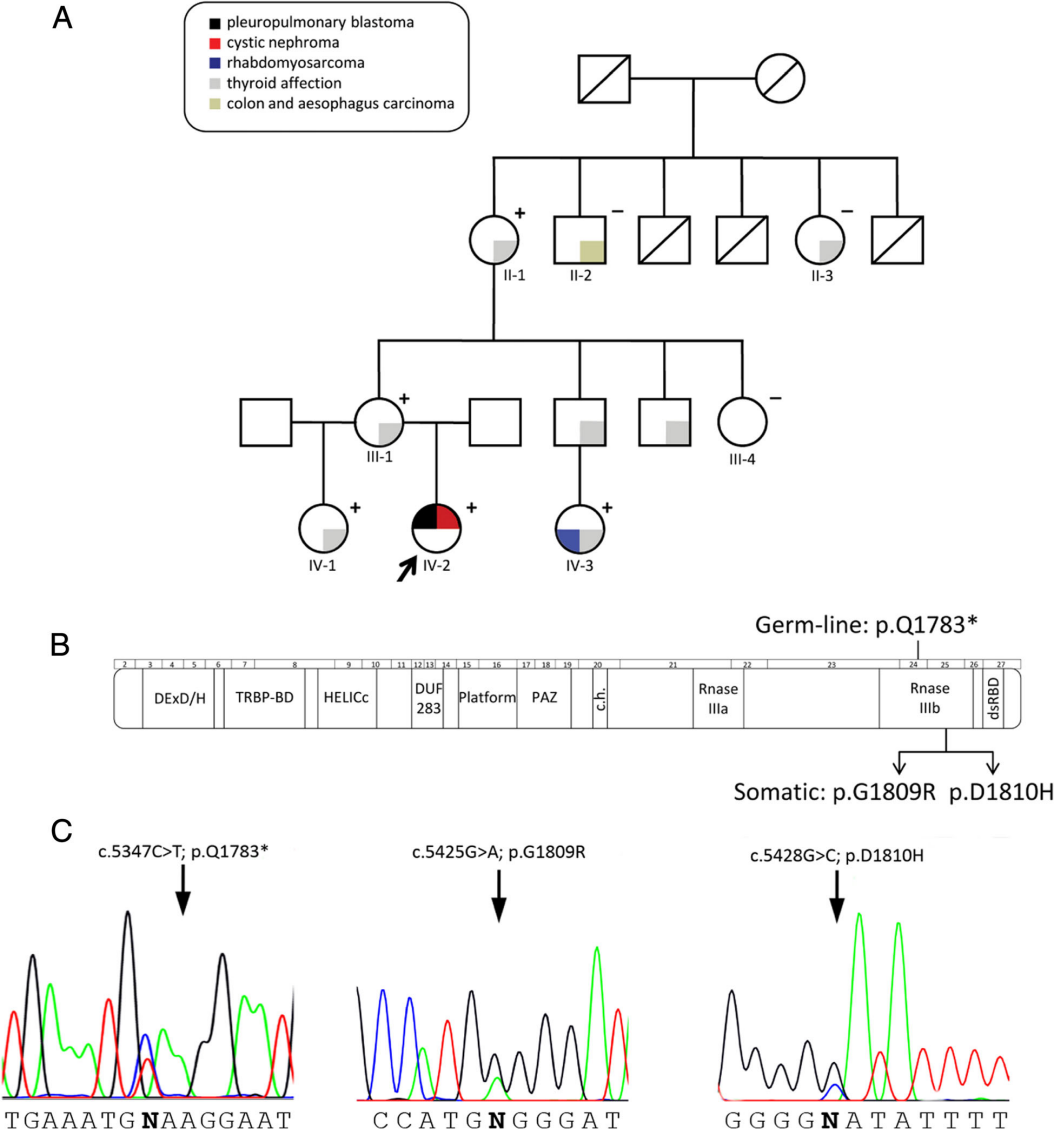
**a**. Pedigree of the studied family. Clinical phenotypes are represented with colors. Individuals screened for *DICER1* mutations are indicated with + or — if the germ-line mutation was present or absent, respectively. The index patient is marked with an arrow. **b**. Schematic representation of DICER1 protein structure and corresponding gene exons. Positions of the here identified mutations are indicated. **c**. Electropherograms corresponding to the germ-line c.5347C > T (p.Q1783*) and somatic c.5425G > A (p.G1809R); c.5428G > C (p.D1810H) mutations

Considering patient’s tumors nature, genetic mutation screening of the complete coding region of the *DICER1* gene in genomic DNA from proband’s blood was performed. It led to identify a nonsense truncating mutation affecting Q1783 residue, codified in exon 24 (c.5387C > T; p.Q1783*). This mutation was found in heterozygosity and predicted to truncate the protein by the RNaseIIIb domain of the enzyme. We studied segregation of this germ-line mutation with diverse pathologies in 7 available relatives and identified the proband’s mother and grandmother as carriers of the mutation (Fig. 2). In addition, a 21-year-old female cousin of the proband who was diagnosed of an embryonal RMS (Fig. 1c) at age 14 and multinodular goiter at age 20, was also germ-line carrier of the *DICER1* mutation. Interestingly, thyroid affection was also reported in most of the family members, being multinodular goiter with calcifications the only remarkable pathological phenotype present in the proband’s mother and grandmother. Of those participating in the study, 4 out of 5 affected of thyroid alterations carried the p.Q1783* mutation.

### Somatic mutation analysis

The presence of an additional somatic mutation in the remaining *DICER1* allele was investigated in genomic DNA obtained from CN and ERMS available tumor samples. Tumor tissue from the PPB was not accessible for molecular studies since the number of tumor cells was very low (Fig. 1b) and the block tissues were exhausted for histological diagnosis. We found a missense heterozygous c.5425G > A (p.G1809R) mutation in DNA obtained from CN, whereas ERMS harbored a c.5428G > C (p.D1810H) substitution, also in heterozygosis (Fig. 2b and c).

Somatic mutational analyses in additional putative oncogenic driver genes with clinical relevance were studied. No alterations in *KRAS, NRAS, EGFR, PIK3CA, BRAF, PDGRFA, TP53, CDKN2A, CDK4, RB1, EGFR, PTEN,* and *MMDM2* genes were detected in the CN. In contrast, in genomic DNA from ERMS we found *TP53* to carry the c.404G > A (p.C135Y) mutation in heterozygosity. ERMS tumor also harbored additional gene alterations: low amplification of *EGFR, PDGFRA,* and *CDK4* genes, and loss of heterozygosity of *CDKN2A* and 19q12-19q13.43 chromosomal regions.

### Mutation allelic configuration

We aimed to investigate if the somatic and germ-line *DICER1* mutations were separate events on different alleles. For this purpose, we analyzed cDNA synthesized from ERMS tumor RNA. Due to the fact that germ-line c.5387C > T (p.Q1783*) mutation creates an *Hpy*CH4V restriction site, we designed a PCR amplification and *Hpy*CH4V digestion assay in which the somatic mutations would be included in the restriction fragment generated by the presence of germ-line mutation. Sequencing analysis of germ-line mutated restriction fragments revealed that germ-line and somatic mutations lied *in trans*.

## Discussion

In this report we identify germ-line and somatic *DICER1* gene mutations in a Caucasian family where two young females were diagnosed, one with cystic nephroma and pleuropulmonary blastoma, and the other with embryonal rhabdomyosarcoma and multinodular goiter. This pedigree demonstrates that genetic alteration in the *DICER1* gene can cause a wide clinical spectrum of carcinomas, in accordance with recent studies [1]. Both affected patients shared the nonsense c.5347C > T germ-line *DICER1* mutation, predicted to truncate the protein, and thus likely impairing activity of the DICER1 enzyme [3].

Interestingly, thyroid affection was also reported in most of the family members. However, we were not able to establish definitive association of the *DICER1* mutation with any specific thyroid disease due to lack of access to detailed clinical histories or affected tissue samples from these patients. As goiter is an endemic disease in Asturias (North of Spain) [4], from where the family is native, it cannot be discarded the existence of further risk factors for thyroid affection within this pedigree.

We identified additional somatic missense *DICER1* mutations in CN and ERMS tumors (p.G1809R and p.D1810H, respectively), affecting metal ion-binding regions of the protein and thus presumably affecting catalytic activity of RNAseIIIb (but not RNAaseIIIa) domain [3]. In contrast with the initially proposed theory of haploinsufficiency of this tumor suppressor gene as the cause underlying DICER1 syndrome [5], our results support the hypothesis that patients harboring a loss-of-function germ-line *DICER1* mutation acquire a second somatic hit during tumorigenesis, resulting in a modified enzyme activity, at the RNAseIIIb level [6]. Loss of RNAseIIIb activity of DICER1 protein has been shown to selectively reduce the generation of 5p miRNAs, with no influence on 3p miRNAs processing, and thus generating a deregulation of control in gene expression [7, 8]. As we have established in our samples, since the second somatic mutation lies *in trans*, both alleles are compromised. This need of a compound disruption of DICER1 may explain the low penetrance of germ-line *DICER1* mutations alone.

Given the wide clinical phenotypes observed, additional somatic gene mutations might be involved in the carcinogenic mechanisms of the syndrome. The malignant nature of ERMS tumor is underlined by the presence of additional genomic alterations, especially *TP53* mutations. The *TP53* gene c.404G > A mutation (p.C135Y) identified in ERMS results in the loss of one of the two p53 intramolecular disulfide bonds. Consequently, the protein conformation and subcellular location are altered [9, 10]. Moreover, it has been proposed that mutant p53 can down-regulate DICER1 expression, enhancing the metastasic potential of tumor cells [11]. Therefore, we suggest that, in addition to *DICER1* loss of full activity, oncogenic cooperation of other genes, such as mutated *TP53*, may involve developing of higher grade tumors. ERMS tumor also harbored genomic alterations in several frequent driver genes.

## Conclusions

Given the variety of tumors developed within this family, and the risk of ovarian cancer (Sertoli-Leydig type) [1, 3, 6] and thyroid affection in adults, a close surveillance might be offered to all at risk family members of DICER1 syndrome pedigrees.

## Abbreviations

CN: Cystic nephroma
ERMS: Embryonal rhabdomyosarcoma
HUCA: Hospital Universitario Central de Asturias
IUOPA: Instituto Universitario de Oncología del Principado de Asturias
MLPA: Multiplex ligation-dependent probe amplification
PPB: Pleuropulmonary blastoma
RMS: Rhabdomyosarcoma
